# RBGO – A journal to support gynecology and obstetrics research in Latin America

**DOI:** 10.61622/rbgo/2024EDT01

**Published:** 2024-03-15

**Affiliations:** 1 Revista Brasileira de Ginecologia e Obstetrícia Brazil Editor-in-Chief of RBGO – Revista Brasileira de Ginecologia e Obstetrícia

In 2016, the Brazilian Federation of the Gynecology and Obstetrics Associations - FEBRASGO began a major restructuring process of the RBGO - *Revista Brasileira de Ginecologia e Obstetrícia* with significant changes in its editorial policy. Since that date, after the editorial board was reformulated, the articles began to be published in English with absolute regularity and the journal was registered in the main international databases. Given the RBGO's commitment to following several aspects of the open science movement, it has become an open access journal and authors are not charged any fees for publishing their articles.

During these eight years, the indicators in its indexing sources were always increasing and, according to data provided by Thieme Publisher (RBGO's partner publisher), from January to October 2023, researchers' access to RBGO's abstracts or full-texts reached the mark of 332,000 and 240,000 respectively, coming from researchers from all over the world, from data analysis by the origin of the access IPs. In 2023, the RBGO achieved Impact Factor 1.2 granted by the Web of Science (WoS) - Journal Impact Factor Trend 2022,^(1)^ and nowadays, it is an internationally recognized journal.

The RBGO stood out in our continent and according to data from the 2022 Scimago Journal & Country Rank, it ranks first among Latin American Gynecology and Obstetrics journals, with SJR 0.29 and H Index 27.^(2)^ These indicators are not very significant when compared with the major journals in Europe and North America. Although Latin American authors actually prefer to send their articles to international journals because of their greater visibility and projection^(3-4)^, the costs of publishing an article in internationally renowned journals of Europe or North America are prohibitive for the vast majority of Latin American researchers. Nowadays, it is not uncommon for journals to charge fees above US$5,000 per article published. For this reason, it is greatly important that Latin America has its own journals edited with quality and internationalized to publicize and give visibility to the research developed here, so that the academic world can have access to its social and scientific reality.

Good quality science, both in basic and application areas, is produced in several research centers in Latin America. In Brazil, postgraduate courses at Master's and Doctorate levels have become the largest source of medical research. Reaching the rest of the world with the product of this science is strategic and in the interest of everyone, particularly the governmental or private bodies and institutions that fund research. Such objectives need, must and can be achieved in line with the new open science policy gaining followers around the world.

Brazil has long occupied a prominent place in global scientific production. In 2022, it ranked 13^th^ in the number of scientific publications, with a production of 78,940 articles published in journals indexed in the WoS.^(5)^ Even though this is an expressive number, it is known that Brazilian scientific production must be much larger than the aforementioned data. Note that the WoS database has a limitation related to its coverage being provided by the WoS Core Collection, its main collection, which is not as comprehensive. It is very focused on publications of the main North American and European journals, containing very little of the science produced and published in Latin America.

In the case of Brazil, the Coordination for the Improvement of Higher Education Personnel – CAPES, a government body that regulates and supervises postgraduate programs, has spent millions of dollars to finance the publications of Brazilian authors in foreign journals. Such resources could be redirected to the development of research itself, since there is a lack of money for this. Why not value Brazilian journals? They have made efforts to become competitive, but need support from the scientific community to send them good quality articles resulting from their academic production and make this a reality. It is a good idea for authors to think so, given that in the current economic situation and its future perspectives, resources from CAPES or other government sources of financing for the publication of articles will certainly be increasingly scarce and the prices of international publications tend to become increasingly higher due to the competitiveness of global scientific production itself.

Considering the good future prospects of the RBGO, we will continue working harder towards its consolidation as the reference journal for gynecology and obstetrics in Brazil and expanding our reach to Latin American researchers. In fact, several Latin American authors have already honored the RBGO with the submission of manuscripts from different areas of activity in gynecology and obstetrics. The RBGO is available for a joint effort with Brazilian and Latin American authors whose support and trust will be fundamental so that together we can also have a quality journal that serves everyone interested in the development of research in Gynecology and Obstetrics in the southern hemisphere.

From this first issue of 2024, the RBGO says goodbye to Thieme Publishers thanking them for their partnership over these eight years. From now on, the RBGO will be edited by a highly qualified technical team and organized by the Editorial Office of FEBRASGO, its owner and maintainer. The RBGO has a new design and can be accessed on its specific website (RBGO Journal), which will offer all information about the journal. From this issue onwards, a continuous flow publication will be adopted, with articles published as they are approved by the editorial board. This is an international trend characterized by agility in publications. The system for submission and evaluation of articles remains the same.
